# Genetic and Anatomical Basis of the Barrier Separating Wakefulness and Anesthetic-Induced Unresponsiveness

**DOI:** 10.1371/journal.pgen.1003605

**Published:** 2013-09-05

**Authors:** William J. Joiner, Eliot B. Friedman, Hsiao-Tung Hung, Kyunghee Koh, Mallory Sowcik, Amita Sehgal, Max B. Kelz

**Affiliations:** 1 Department of Pharmacology, University of California San Diego, La Jolla, California, United States of America; 2 Department of Neuroscience, Howard Hughes Medical Institute, University of Pennsylvania, Philadelphia, Pennsylvania, United States of America; 3 Center for Sleep and Circadian Neurobiology, University of Pennsylvania, Philadelphia, Pennsylvania, United States of America; 4 Department of Neuroscience, Thomas Jefferson University, Philadelphia, Pennsylvania, United States of America; 5 Department of Anesthesiology and Critical Care, University of Pennsylvania, Philadelphia, Pennsylvania, United States of America; University of California, San Francisco, United States of America

## Abstract

A robust, bistable switch regulates the fluctuations between wakefulness and natural sleep as well as those between wakefulness and anesthetic-induced unresponsiveness. We previously provided experimental evidence for the existence of a behavioral barrier to transitions between these states of arousal, which we call neural inertia. Here we show that neural inertia is controlled by processes that contribute to sleep homeostasis and requires four genes involved in electrical excitability: *Sh*, *sss*, *na* and *unc79*. Although loss of function mutations in these genes can increase or decrease sensitivity to anesthesia induction, surprisingly, they all collapse neural inertia. These effects are genetically selective: neural inertia is not perturbed by loss-of-function mutations in all genes required for the sleep/wake cycle. These effects are also anatomically selective: *sss* acts in different neurons to influence arousal-promoting and arousal-suppressing processes underlying neural inertia. Supporting the idea that anesthesia and sleep share some, but not all, genetic and anatomical arousal-regulating pathways, we demonstrate that increasing homeostatic sleep drive widens the neural inertial barrier. We propose that processes selectively contributing to sleep homeostasis and neural inertia may be impaired in pathophysiological conditions such as coma and persistent vegetative states.

## Author Summary

An annual 234 million surgical procedures are performed worldwide, making general anesthetics among the most common drugs administered to humans. Remarkably, however, we still do not understand the mechanisms by which general anesthetics render patients unconscious or the processes that re-establish consciousness upon emergence from anesthesia. We previously showed that the brain resists transitions between the wakeful and anesthesia states by generating a barrier to such transitions in both directions. We also showed that the existence of this barrier is conserved from invertebrates to mammals. In our present work, we use the genetic tractability and the simplified nervous system of the fruit fly *Drosophila melanogaster* to show that four genes are required to maintain this barrier. We also show that, as in mammals, there is overlap between pathways regulating natural sleep and general anesthesia. We propose that some of these shared pathways are impaired in conditions such as coma and persistent vegetative states, in which the barrier to transitioning to the waking state appears to be insurmountable.

## Introduction

Inherent in the design of robust and bistable switches is hysteresis, which prevents small or random fluctuations from triggering a state change in the system [1]. Arousal states display bistable behavior and are regulated by a biologic switch that possesses hysteretic properties [2]–[5]. Inhaled general anesthetics offer the opportunity to study the molecular and neuroanatomical pathways essential for the aroused, conscious state as well as the orderly transition to and from the unconscious state [6], [7]. General anesthetics are known to exert their hypnotic properties in part by interacting with endogenous systems that regulate arousal state [8]–[10]. Functionally these interactions include modulation of ion channels to suppress neuronal excitability [11]. Behaviorally the effects of these interactions are described by various endpoints that correspond to different depths of general anesthesia including (in order) amnesia, hypnosis, and ultimately immobility [12]. Although historically most studies of anesthetics have been performed on mammals, similar endpoints have been described for invertebrates. Furthermore, in vertebrates and invertebrates similar concentrations of anesthetics induce those endpoints [13]. Phylogenetically and functionally related classes of genes also alter anesthetic sensitivity across multiple phyla [7], [14]–[16]. Collectively these data suggest that mechanisms of arousal control have been conserved throughout evolution, even if gross brain anatomy has diverged.

We previously established in both mice and fruit flies that different concentrations of anesthetics are required for induction of and emergence from general anesthesia, and that this hysteresis cannot be explained solely by pharmacokinetics [7]. Hysteretic dissociation of anesthetic induction from emergence is consistent with the existence of a barrier termed “neural inertia” that separates and stabilizes behavioral states. The inertial barrier leads to maintenance of wakefulness or anesthesia, and presumably exists to oppose rapid and potentially catastrophic transitions between these states. The effective size of the inertial barrier can be estimated by measuring the area between the induction and emergence curves. Switching between wakeful and anesthetized states would thus be difficult with high neural inertia but would occur easily with low neural inertia. Here we sought insight into the mechanisms underlying this behavioral state barrier by studying its genetic and anatomical bases as well as its relation to other arousal-regulating processes such as circadian clock function and sleep.

Previous studies have demonstrated that the concentration-response curve for induction of anesthesia can be manipulated genetically, particularly by mutations that alter excitability [7], [17]. In the present study we demonstrate that the inertial barrier can be collapsed by loss-of-function mutations in genes that have opposing effects on induction of isoflurane anesthesia. These genes encode the hyperpolarizing Shaker potassium channel (Sh) and its positive modulator SLEEPLESS (SSS), the loss of which causes resistance to anesthesia induction, as well as the depolarizing cation channel, narrow abdomen (NA) and its positive modulator UNC79, the loss of which increases sensitivity to anesthesia induction. The requirement of all four genes for maintenance of neural inertia by isoflurane is consistent with a model in which these genes contribute to mutual inhibition by arousal-promoting and arousal-suppressing loci to create a bistable system in which either the waking or anesthetized state predominates, similar to the “flip-flop” switch that has been proposed to stabilize waking and sleep in mammals [2]. Indeed, we find that the *sss* gene acts in different sets of neurons to influence induction of and emergence from anesthesia. We also find that arousal *per se* does not control neural inertia since the inertial barrier is unaffected by certain hyperaroused mutants. Instead, as in previous studies with other anesthetics [18]–[20] we report that emergence from anesthesia becomes more difficult in sleep-deprived animals. Consequently, the neural inertial barrier to reversing the anesthetized state is broadened with sleep deprivation. Collectively our data suggest that some molecular and anatomical arousal pathways that underlie sleep homeostasis also contribute to neural inertia.

## Results

### Induction and emergence contribute to neural inertia by distinct genetic mechanisms

**Figure 1:**
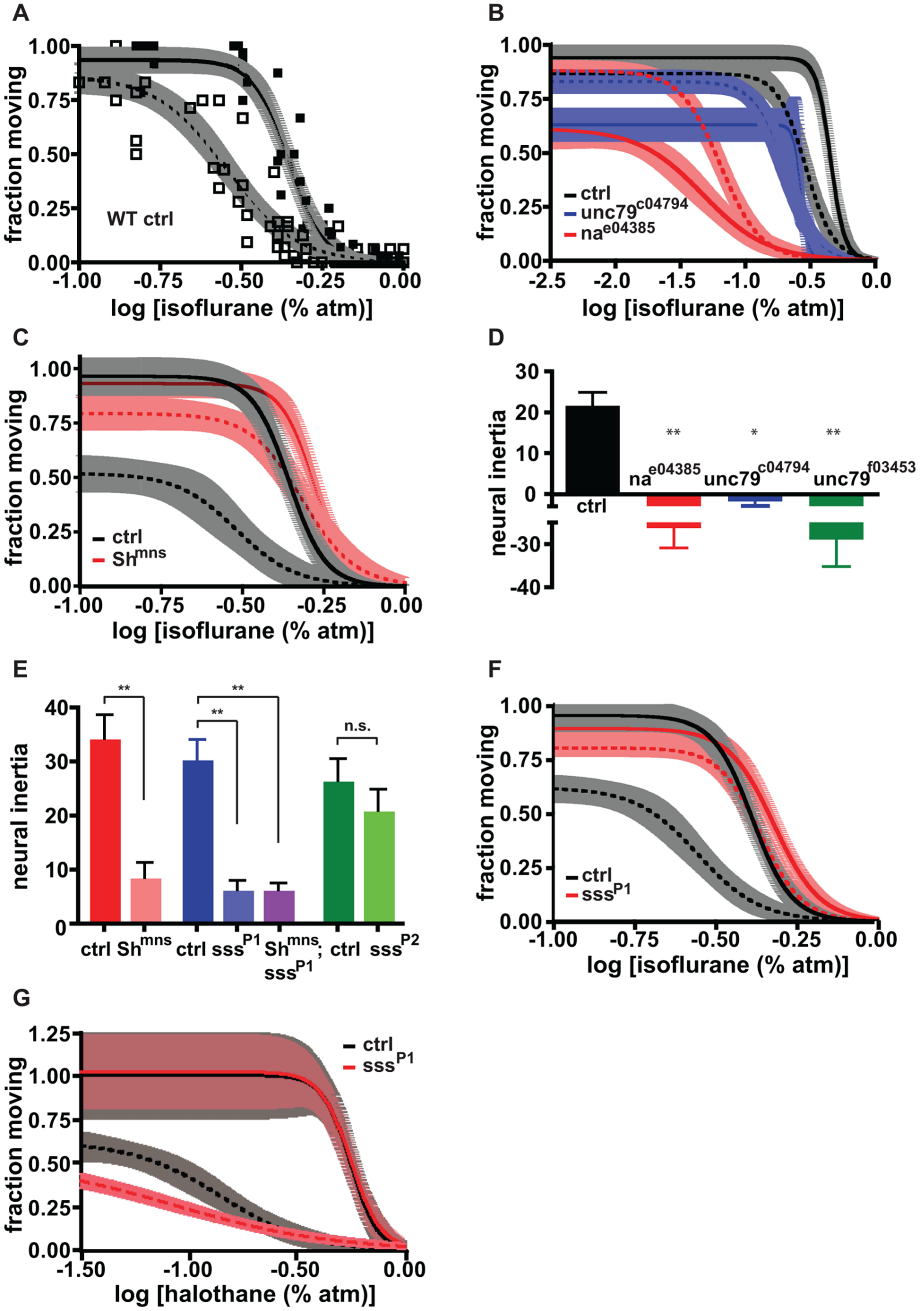
Induction and emergence contribute to neural inertia by distinct genetic mechanisms. (a) Dose-response curves for induction of (solid curve) and emergence (dashed curve) from isoflurane-dependent anesthesia exhibit hysteresis. Shaded areas represent 95% confidence intervals for Hill fits to data points for induction (solid) and emergence (open symbols). (b) Loss-of-function mutations in *na* (red) and *unc79* (blue) increase sensitivity to induction of anesthesia (solid curves) without causing an equivalent shift in emergence curves (dashed curves) relative to controls (black). (c) The *mns* mutation of the *Sh* gene decreases sensitivity to induction of anesthesia (solid red) and causes a disproportionate shift in the emergence curve (dashed red) relative to controls (solid and dashed black lines). (d) Neural inertia is collapsed in *na* and *unc79* mutants in which CNS excitability is thought to be reduced. (e) Neural inertia is collapsed in strong loss-of-function alleles of *Sh* and *sss*, but not in a weak allele of the latter. (f) As in *Sh^mns^*, the *sss^P1^* mutation decreases sensitivity to induction of anesthesia (solid red) and causes a disproportionate shift in the emergence curve (dashed red) relative to controls (solid and dashed black lines). (g) Induction (solid) and emergence (dashed) curves for halothane-dependent anesthesia are relatively unaffected in *sss^P1^* mutants (red) compared to controls (black). n.s., not significant compared to control, * p<.01, ** p<.001 by one-way ANOVA with post-test Bonferroni correction for multiple comparisons.

We undertook the present study to determine whether distinct mechanisms control induction of and emergence from anesthesia. To establish baseline levels of hysteresis for wildtype animals we first established dose-response curves for induction and emergence using isoflurane. As in mammals [7] the two curves are distinct in flies (Figure 1a), suggesting that induction and emergence are not caused by identical processes operating in reverse. However, unlike mammals some flies do not resume movement during the stepwise, downward anesthetic titration. These animals are not dead, but rather exhibit a slower pattern of emergence not amenable to plotting on this time scale (Figure 1b, c, f). The failure of a *Drosophila* population to fully emerge when anesthetic levels are reduced below the limit of detection is a property subject to genetic regulation and consequently contributes to the measurement of neural inertia [7].

Next we examined induction and emergence curves for animals bearing lesions in genes that have previously been implicated in anesthetic sensitivity. In agreement with published studies [16], [21], [22] we found that disruption of *na* dramatically increased sensitivity to induction of the anesthesia state by isoflurane, as did disruption of *unc79*, a gene that is believed to act in the same pathway (Figure 1b). Since wildtype NA is thought to underlie a leak sodium current that promotes excitability [23], we asked whether the correlation between change in excitability and anesthesia induction would apply to other genes that regulate excitability. We began by examining the contribution of Shaker (Sh) potassium channels, which decrease excitability, and confirmed our recent finding that a loss of function mutation in *Sh* decreases sensitivity to induction (Figure 1c).

The phenotypes of animals bearing mutations in *na*/*unc79* and *Sh* suggest that excitability is positively correlated with resistance to induction of isoflurane anesthesia. The *Sh* mutation increases excitability and also increases resistance to induction of anesthesia by isoflurane. We hypothesized that a similar positive correlation would exist between excitability and ease of emergence from isoflurane anesthesia. Indeed, *Sh* mutants readily emerged from anesthesia. In fact, in these flies emergence is impacted much more than induction and occurs at relatively high concentrations of isoflurane, thereby leading to a collapse of neural inertia (Figures 1c,e). The same reduction in neural inertia can be observed for animals with disrupted expression of the *sleepless* (*sss*) gene, which positively regulates Sh K channels [24], [25]. Like *Sh* mutants, *sss* mutants show resistance to anesthesia induction (Figure 1f). And as with *Sh* mutants, the emergence curve for strong *sss* mutants is compressed against the induction curve, leading to a collapse of neural inertia (Figures 1e,f). The ability of *sss* mutants to reduce the neural inertial barrier is correlated with the strength of the underlying mutation. *sss^P1^* mutants, with no detectable SSS protein, have a more extreme phenotype than hypomorphic *sss^P2^* mutants in which SSS expression is reduced by ∼30% (Figure 1e, Figure S1a and [25]).

However, a surprising result arises from analysis of *na*/*unc79* mutants. Although these mutants have decreased excitability and therefore would be predicted to resist emergence from anesthesia, they exit the anesthetized state at doses of isoflurane similar to or greater than those required for induction. Thus, *na/unc79* mutations reduce the barrier to changing behavioral states in both directions (Figures 1b,d). That is, they promote transitions from the aroused to the anesthetized state and also from anesthesia back to the aroused state. Consistent with this observation, *na* mutants have highly fragmented bouts of waking and sleep (Figure S2a).

*sss* is known to regulate Shaker K channels [24], [25], so we combined *sss* and *Sh* mutants to determine if the two genes act in the same pathway to affect neural inertia. Consistent with this interpretation, the EC_50_ for induction in *Sh;sss* double mutants was similar to or only slightly higher than that in *Sh* or *sss* single mutants (Figure S1b–d; Table S1). We also found that *Sh* loss of function heterozygotes have reduced neural inertia, whereas *sss^P1^* heterozygotes do not, indicating that anesthetic sensitivity is more responsive to reductions in *Sh* than in *sss* (Figure S1e).

Having determined that anesthesia induction and emergence are controlled by different genes, we next asked whether different types of anesthetics act on the same or different arousal-regulating pathways. To address this question, we measured dose-response curves for induction and emergence in the presence of halothane, another common volatile anesthetic, using both wildtype and *sss^P1^* mutants. As with isoflurane, halothane exposure revealed a neural inertial barrier between the awake and anesthetized states in control animals. In contrast to what was observed with isoflurane, however, the halothane induction curve was unaffected and the emergence curve was slightly left-shifted in *sss^P1^* mutants, leading to expanded neural inertia (Figure 1g). The failure of isoflurane and halothane to elicit qualitatively similar shifts in induction and emergence in *sss* mutants is consistent with published reports suggesting different anesthetics act on different molecular or neuroanatomical pathways [26], [27].

### Different brain regions mediate effects of *sss* on anesthesia-sensitive arousal

The neural pathways underlying the actions of volatile anesthetics are not well understood in mammals, and in invertebrates even less is known. Progress has been stymied in part by an inability to identify and study the roles of the different circuits that control arousal, each of which may be affected to different degrees by a given anesthetic. Our ability to collapse neural inertia with mutations that have opposing effects on isoflurane induction suggests that induction can be genetically dissociated from processes that stabilize the anesthetized state and prevent emergence from it (Figures 1b–g). Genetic dissociation of neural inertia and anesthesia induction raises the possibility that these phenomena may also be anatomically separable.

**Figure 2:**
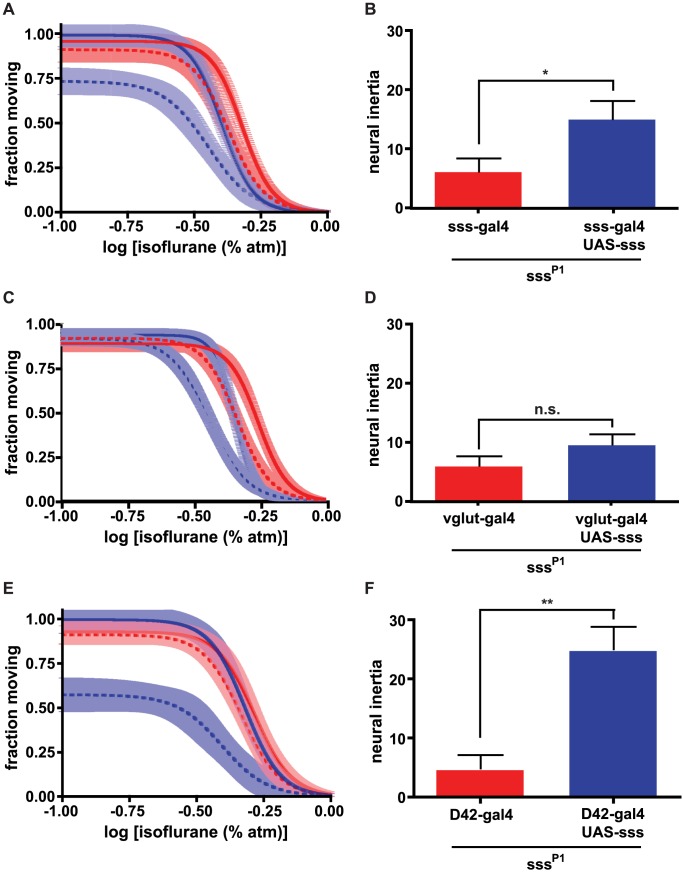
Neuroanatomic dissociation of neural inertia and anesthesia induction is unmasked by restoration of *sss* expression in different regions of the brain. (a, b) Expression of *sss* behind the *sss* promoter rescues altered anesthesia induction and emergence of *sss^P1^* mutants, leading to a rescue of neural inertia. (c,d) Expression of *sss* behind the *vglut* promoter rescues altered anesthesia induction but not neural inertia. (e,f) Expression of *sss* behind the D42 promoter rescues altered anesthesia emergence as well as neural inertia but not induction in *sss^P1^* mutants. In all panels, induction and emergence curves are shown as solid and dashed lines, respectively. In each case, promoter-GAL4/+ (red) and promoter-GAL4/+;UAS-*sss*/+ (blue) animals were generated in a *sss^P1^* background. n.s., not significant, * p<.05, ** p<.001 by unpaired two-tailed t-test.

Because sleep phenotypes of *sss* mutants are effectively rescued by localized expression of a *sss* transgene, we used this approach to determine if the induction and neural inertia phenotypes of *sss^P1^* mutants arise from distinct anatomic loci. We coupled various promoters driving the GAL4 transcription factor to a transgene encoding wildtype *sss* in a homozygous *sss^P1^* mutant background, then determined correlations between expression patterns and rescue of the two *sss^P1^* phenotypes: (a) right-shifting of induction and (b) a more dramatic right-shifting of emergence with consequent collapse of neural inertia. As expected, the native *sss* promoter rescued these phenotypes robustly (Figure 2a,b). SSS expression is high in the head and particularly in the brain compared to the body [25], so we asked whether *sss* expression in the nervous system is sufficient to regulate transitions between the anesthesia and waking states. Importantly, the pan-neuronal driver *elav*-GAL4 rescued induction, emergence, and neural inertia whereas the glial driver *repo*-GAL4 had no effect on these phenotypes (Figures S3a–c). These results are consistent with the idea that a barrier between the waking and anesthetized states is generated by neurons in the brain.

Another driver, *vglut*-GAL4, which expresses in glutamatergic neurons, phenocopied the rescue of the induction phenotype observed with *sss*-GAL4 in a *sss^P1^* mutant background (Figure 2c; Table S1). Restoring wildtype SSS protein to glutamatergic neurons also significantly altered the EC_50_ for emergence (Table S1), shifting the emergence dose-response curve roughly 20%, in parallel with the induction rescue. However, unlike the *sss* promoter, the *vglut* promoter could not rescue the collapse of neural inertia in *sss^P1^* mutants (Figure 2d). Importantly, this result illustrates that glutamatergic expression of *sss* is insufficient to restore the barrier between the waking and anesthetized states. Unlike *vglut*, another promoter, D42, failed to rescue the induction phenotype of *sss^P1^* mutants. However, restoration of *sss* expression in D42-expressing neurons of *sss^P1^* mutants rescued the concentration-response curve for emergence, leading to wildtype levels of neural inertia (Figures 2e,f). Together, the results of rescuing the *sss^P1^* anesthesia phenotypes with *vglut*-GAL4 and D42 suggest that different sets of neurons are involved in entry into, as well as exit from and stabilization of, the anesthetized state.

Promoters with broad expression patterns such as *cha* and C309 rescued both induction as well as emergence to varying degrees. For emergence, significant partial or full rescue was observed with *cha*-GAL4, MZ1366, Mai301, Sep54, 30y and C309. However, neural inertia was only rescued by a subset of these promoters, namely Mai301, Sep54 and 30y. Importantly, induction was not rescued by any of these drivers. Moreover, the majority of drivers failed to alter any phenotype (Figures S3a–c). These data suggest that large but divergent populations of neurons separately control induction and emergence and consequently the stability of the anesthesia state, although we cannot exclude the possibility that small subsets of cells labeled by the positive drivers are responsible for the rescue.

### Neural inertia is controlled by arousal mechanisms shared by sleep homeostasis

**Figure 3:**
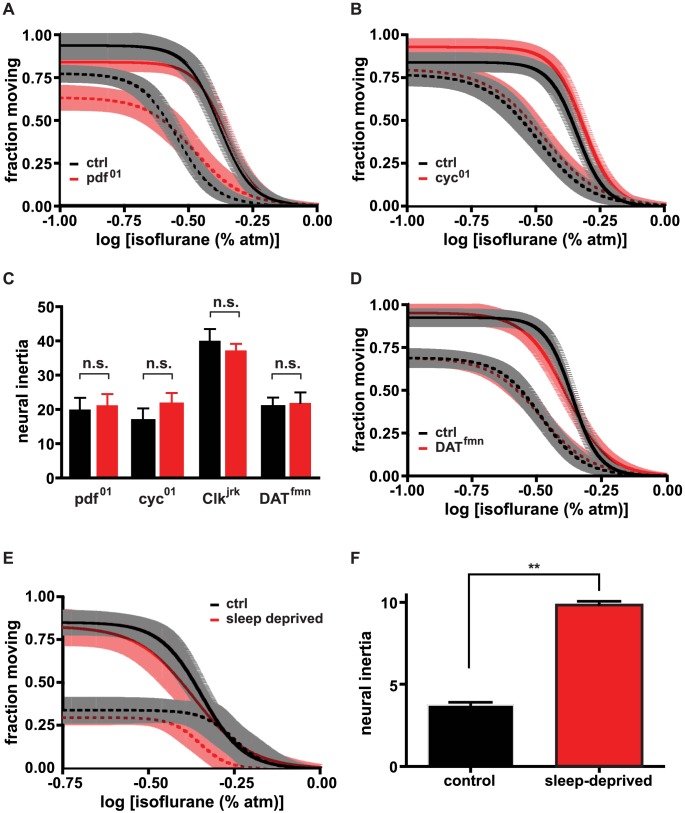
Neural inertia is affected by sleep homeostasis but not by mutations that exclusively impair non-homeostatic (baseline) sleep control or circadian clock function. (a) Induction (solid) and emergence (dashed) curves for *pdf^01^* mutants (red) and controls (black). (b) Induction (solid) and emergence (dashed) curves for *cyc^01^* mutants (red) and controls (black). (c) Measurements of neural inertia do not vary significantly between *pdf^01^*, *cyc^01^*, *Clk^jrk^*, *DAT^fmn^* mutants (red) and their respective sibling controls (black). (d) Induction (solid) and emergence (dashed) curves for *DAT^fmn^* mutants (red) and controls (black). (e,f) Dose-response curve for anesthesia emergence is left-shifted (dashed) without a change in anesthesia induction (solid) following 24 hrs sleep deprivation (red, dep; black, control), leading to an increase in neural inertia. n.s., not significant compared to control (one-way ANOVA with post-test Bonferroni correction), ** p<.001 by unpaired two-tailed t-test.

Anesthesia and sleep may both involve suppression of arousal [9], [10], an idea that is supported by the effects of mutations in *Sh* and *sss* on these behavioral states [7], [24], [25], [28]. We next addressed whether anesthesia and sleep are regulated by similar biological processes. Sleep drive has been modeled as the combined output of the circadian clock and a homeostatic process of unknown composition [29]. To test whether the same processes modulate the arousal circuitry affected by isoflurane we first attempted to measure concentration-response relationships at different times of day. Measuring the transition from the awake to the anesthetized state in our assay requires that animals be active prior to exposure to drug. This waking activity could not be achieved during long time periods including ZT3-9 and ZT14-22 since at these times animals have a high probability of being immobile due to their natural propensity to sleep. Thus, we addressed circadian regulation by assaying effects of circadian clock mutants. We restricted all measurements described herein to ∼2 hrs starting just after ZT10, near one of the two daily peak activity times. During this period we addressed the circadian contribution to anesthetic sensitivity using a mutant in which the output signal from the clock is abolished, *pdf^01^*, and two core clock mutants, *cyc^01^* and *Clk^jrk^*. We found that the induction and emergence profiles, and hence neural inertia, were unaffected in all three mutants (Figures 3a–c; Figure S4a), indicating that the circadian clock is not required for isoflurane-dependent anesthesia.

In addition to abolishing circadian clock cycling, *cyc^01^* and *Clk^jrk^* mutations cause reductions in sleep [30], [31], much like *Sh* and *sss* loss of function mutations [25], [28]. *Sh* and *sss* mutants, however, display both sleep and isoflurane anesthesia phenotypes, whereas *cyc* and *Clk* mutants do not exhibit the latter. We wondered how common it is to find mutations like *cyc^01^* and *Clk^jrk^* that lead to dissociation of the anesthesia and sleep phenotypes. It has been suggested that general anesthetics co-opt arousal pathways that have evolved to regulate the sleep/wake cycle [9], [10]. We thus hypothesized that anesthesia involves an overlapping set, or even a subset, of arousal pathways normally utilized to regulate sleep. If this were the case then non-circadian mutants might also be identifiable that reduce sleep without affecting the anesthetized state. To test this hypothesis, we examined the effects of *DAT^fmn^* mutants, which have impaired dopamine transporter function, on the concentration-response relationships of induction of and emergence from isoflurane-dependent anesthesia. Like *cyc^01^* and *Clk^jrk^* mutants, *DAT^fmn^* mutants show normal anesthetic sensitivity but abnormally low sleep (Figures 3c,d; Figure S4b, and [30]–[32]). Thus, not all arousal pathways are shared between sleep and anesthesia.

*cyc^01^*, *Clk^jrk^*, *Dat^fmn^ Sh^mns^* and *sss^P1^* reduce daily sleep, and we show here that a mutation in *na* causes an increase in sleep as well as fragmentation of sleep and wake bouts (Figure S2a,b). Thus, all these mutations alter levels of daily sleep, but only *sss* mutants are known to reduce sleep homeostasis, the process that promotes sleep in response to prolonged wakefulness. To address directly whether the homeostatic component of sleep contributes to the response to anesthesia, we tested whether sleep deprivation could alter sensitivity to isoflurane. In wildtype animals, 6–24 hrs of sleep deprivation elicits robust homeostatic recovery sleep [25], [33], a reflection of increased sleep drive and depressed arousal. We exposed experimental animals to mild mechanical agitation for 24 hrs, up to and including times at which animals were treated with isoflurane. Control animals were similarly agitated only during isoflurane treatment and for 15 minutes beforehand. We have previously observed that such agitation is sufficient to awaken sleeping flies but not those that are anesthetized. Consistent with the hypothesis that the anesthesia state may use pathways underlying sleep homeostasis, we found that increasing homeostatic sleep drive led to a small but significant shift in the EC_50_ for emergence. Although no change was observable in the EC_50_ for induction of the anesthesia state relative to controls, the net effect was a significant increase in neural inertia for sleep-deprived animals (Figures 3e,f; Table S1).

## Discussion

We previously demonstrated an evolutionarily conserved property of the brain, resistance to changes in arousal state, which we have termed neural inertia [7]. One hallmark of this observed phenomenon, hysteresis of anesthetic action, has been described in mathematical simulations of cortical activity in response to anesthetics as well [5], [34]. In these models and in various biological systems, bistability and ultimately feedback are required for hysteresis. By bistability we mean that a system can exist in either of two stable states. In our case these are the anesthetized and waking states. Other examples of bistability abound in nature, such as metabolic adaptations [1], [35], [36] and cell fate decisions [1], [37]. In these situations, changes in concentration of a biochemical signal lead to positive or negative feedback, resulting in a subsequent change in sensitivity to the initial signal. Consequently, exit from the particular state must proceed along a different concentration-response curve than led to entry into the state.

Another way to think about bistability is in terms of state diagrams. In the simplest example, an inducer (a drug in our case) provides the binding energy to initiate the transition from the awake state to a state of anesthesia. Once the transition is complete and the state change has occurred, a feedback mechanism is initiated that increases the sensitivity of the system to the drug, thus requiring an even greater opposing shift in concentration of drug to reverse the process. Feedback can come at the single cell level, as we have outlined above, but it can also derive from recruitment of other cell types into a unified circuit. A relevant example of this phenomenon can be found in the mutual excitation of thalamic and cortical neurons required for waking. Excitation of thalamic nuclei by arousal systems leads to a switch from the burst firing state characteristic of sleeping or anesthesia to the tonic firing state characteristic of waking [38], [39]. The result is recruitment of cortical neurons into a positive feedback loop that maintains excitation of both sets of neurons, thus stabilizing the waking state.

It has been hypothesized that anesthetics recruit sleep circuitry, perhaps by suppressing arousal systems [9], [10]. But what is the nature of this circuitry? One possibility is that anesthetics could act on a bidirectional neuronal pathway that regulates both induction and emergence. In this scenario, initial anesthetic exposure would alter activity in the pathway such that upon emergence, the population would behave differently and thus produce hysteresis. Alternatively, anesthetics could affect two separate (or partially non-overlapping) pathways: one whose function is disrupted to permit induction and a second whose function must recover to permit emergence. We cannot say for certain where general anesthetics such as isoflurane or halothane act in the fly brain. However, we find that different drivers can separately rescue the shifts in induction and emergence caused by the *sss^P1^* mutation. Thus, our results support a role for distinct anatomical circuits in control of bistability of the waking and unconscious states. Notably, neural inertia is distinct from sensitivity to induction of the anesthesia state since we can collapse hysteresis both with mutations that profoundly inhibit and those that facilitate induction of anesthesia. Most strikingly, *na/unc79* mutations facilitate induction of anesthesia, which might be predicted based upon their decreased neural excitability. But they also promote emergence from anesthesia, indicating that they more generally destabilize behavioral states. *na* mutants also show frequent transitions between sleep and waking (i.e. fragmentation of sleep and wake bouts) and provide perhaps the best genetic evidence for the existence of molecules that stabilize behavioral states.

**Figure 4:**
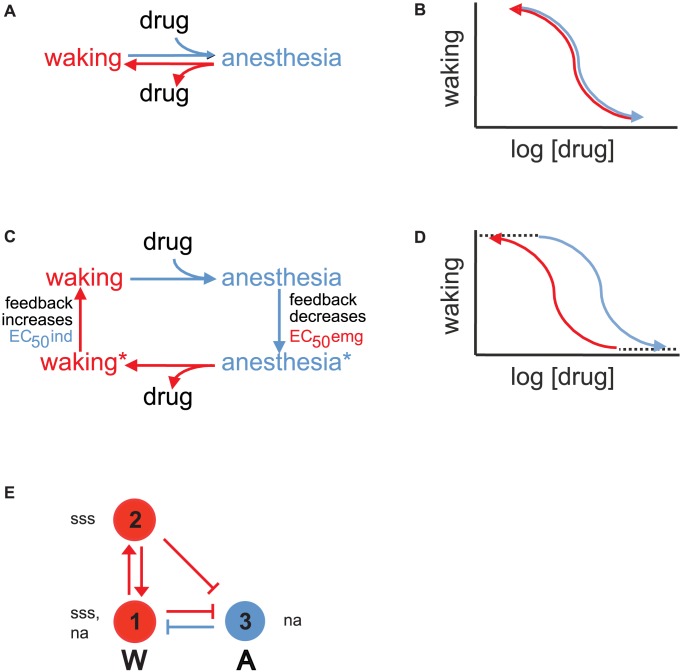
Models of anesthesia depend on feedback underlying bistability. (a) Simple kinetic model describing drug-dependent behavioral state changes in the absence of bistability. (b) In the absence of feedback and bistability, dose-response curves for anesthesia induction and emergence are independent of history of prior behavioral state and thus coincide. (c) Addition of feedback upon binding or unbinding of drug leads to additional, more stable anesthesia and waking states. (d) Feedback and bistability lead to hysteresis in dose-response curves for anesthesia induction and emergence. (e) Three cell circuit model underlying bistability of waking and anesthesia in *Drosophila*. Arrows and perpendicular bars indicate feedforward excitation and inhibition, respectively. Red cells promote and blue cells inhibit waking (W). Cells 1 and 2 express *sss* and are excited by loss-of-function mutations in this gene, whereas cells 1 and 3 express *na* and are inhibited by loss-of-function mutations in this gene. For the sake of simplicity, *Sh* and *unc79* have been omitted but may be coexpressed with *sss* and *na*, respectively.

Collectively our findings suggest the existence of certain features of a minimal neural circuit that underlies neural inertia. First, components must exist to stabilize the waking vs the anesthesia state. This requirement is illustrated in the following example. In the absence of bistability, a simple kinetic model describes the transitions between two states, one unbound and the other bound to drug (Figure 4a). The resulting dose-response curves for the forward and reverse reactions are coincident (Figure 4b). In a bistable situation such as waking and anesthesia, we propose that upon entry into either state, distinct feedback mechanisms are activated to shift drug sensitivity toward stabilization of the state (Figure 4c). As a result the dose-response curves for induction and emergence show hysteresis (Figure 4d). At a circuit level, feedback could take the form of mutual inhibition or positive reinforcement by neurons that facilitate each state (Figure 4e).

Next, we can assign additional components based on measured effects of mutations on induction and emergence. Since loss of excitatory NA facilitates both entry into anesthesia (induction) and exit from this state (emergence), we suggest that *na/unc79* is expressed in both arousal-promoting and arousal-inhibiting cells (Figure 4e). If *Sh/sss* were expressed in the same neurons, mutations in these genes should have opposing effects to those in *na/unc79*. However, while mutations in *Sh/sss* retard entry into anesthesia, they do not retard exit from this state. Thus, we place *Sh/sss* in arousal-promoting but not arousal-inhibiting cells (Figure 4e).

Lastly, there appear to be at least 2 subpopulations of neurons that have distinct effects on induction and emergence when *sss* is present. Thus, we divide the arousal-promoting portion of our circuit into two parts that reinforce each other's activity as well as suppress the arousal-inhibiting side of the circuit (Figure 4e).

Now we can assess how well our simple 3-cell model explains our data (Figure 4e). During isoflurane anesthesia, activity in the wake-suppressing side of the circuit (blue, A) dominates. Once activated, A cells impede emergence by inhibiting the wake-promoting system (red, W). As a result, exiting the anesthetized state requires that anesthetic be lowered substantially below the level required to enter this state. This effect is responsible for the leftward shift of the emergence curve relative to the induction curve (contrast Figure 4b with Figure 4d).

During waking the situation reverses. Activity within W cells dominates and is stabilized by mutual reinforcing connections (red vertical arrows). This positive feedback increases the amount of anesthetic required to overcome the waking state and induce anesthesia. This effect leads to a rightward shift of the induction curve relative to the emergence curve in Figures 4b,d. Additional stability in the waking state is provided by inhibition of the A cells.

This model also explains the effects of our mutants. We propose that loss of *na* in cell 1 leads to reduced activity in the W circuit, thus left-shifting the induction curve. We also propose that loss of *na* in cell 3 leads to reduced activity in the A circuit, thus right-shifting the emergence curve. The net effect is collapse of hysteresis. For *sss* mutants we propose that activity is increased in cells 1–2 of the W circuit, which results in two changes. The first is a right-shift of the induction curve. The second is inhibition of the A circuit even during anesthesia, which destabilizes this state and right-shifts the emergence curve. Again, the net effect is collapse of hysteresis.

Our model also explains how restoration of *sss* expression in distinct cells can rescue the induction, emergence and neural inertia phenotypes of *sss* mutants. We propose that *sss* in cell 1 reduces suppression of the A side of the circuit during waking, thus restoring the position of the right-shifted induction curve. In contrast, *sss* in cell 2 reduces suppression of the A side of the circuit during anesthesia, thus restoring the position of the right-shifted emergence curve.

We have also addressed a long-standing hypothesis about the means by which anesthetics are thought to modulate arousal - that is, by co-opting existing sleep-regulatory mechanisms [9], [10]. We have demonstrated that of 8 genes we tested that have been reported to contribute to control of baseline (daily) sleep in flies, only a subset affect induction and stability of isoflurane-dependent anesthesia. Among the genes that have no effect are 3 that are essential to timekeeping by the central circadian clock, suggesting that circadian control of arousal is not required for normal isoflurane sensitivity. Similarly, reduced dopamine transporter function does not affect induction of or emergence from isoflurane-dependent anesthesia, despite leading to a profound reduction in sleep.

If these distinct arousal pathways do not contribute to circuits underlying anesthesia, then which ones do? A recent study suggests that dopaminergic inputs to the fan-shaped body contribute to sensitivity to isoflurane anesthesia, but this study did not distinguish between effects on induction and emergence [40]. Notably we find that D42-driven expression of *sss*, which rescues altered emergence and neural inertia but not induction in *sss^P1^* mutants, does not appear to express in the fan-shaped body [24], so it is likely that other neurons contribute to the circuitry underlying isoflurane anesthesia as well. D42 is a promoter that is known to express in mixed populations of central neurons as well as some neurons of the peripheral nervous system [24]. D42 was derived from an enhancer trap screen, rather than a cloned gene regulatory element, and the site of insertion of its Gal4-containing P-element is unknown. Thus, the fly gene that it is associated with and any corresponding mammalian gene, including the neurons that express the latter, are also unknown. Due its broad expression pattern, it is difficult to say which neurons are mediating the effects of the D42 driver. However, one possibility is the mushroom bodies, where D42 is known to express [24] and which we have previously shown to participate in sleep regulation [41].

Like our own work, several studies also indicate that mechanisms underlying sleep homeostasis may contribute to the anesthetized state [18]–[20] (though unlike ours, these studies suggest that sleep deprivation impacts both induction and emergence). Consistent with this hypothesis, we find that elevated homeostatic pressure to sleep suppresses arousal and increases neural inertia. This hypothesis is also supported by our finding that *sss^P1^* mutants, which show reduced sleep homeostasis, exhibit reduced neural inertia. This effect is likely to be confined to specific brain circuitry since the promoters that rescue collapsed neural inertia represent a subset of the promoters that rescue sleep loss in *sss* mutants [24]. However, our hypothesis does not explain why mutants such as *cyc^01^*, *Clk^jrk^* and *DAT^fmn^* have normal neural inertia. These mutants sleep substantially less than controls [30], [31], [32] and thus might be expected to have accumulated homeostatic drive to sleep. We hypothesize that these two effects - reduced sleep and increased sleep drive - counteract each other in terms of neural circuit activity, thus leading to no net effect on isoflurane sensitivity. In contrast, in the absence of intact sleep homeostatic mechanisms, such as we find in *sss* mutants [25], the resulting imbalance in neural circuit activity unmasks changes to the induction and emergence processes. To extend this hypothesis further, mutations that alter induction, emergence or neural inertia may lead to the identification of genes that contribute to sleep homeostasis.

Interestingly, the relationship between sleep homeostasis and neural inertia cannot necessarily be generalized to all anesthetics. Indeed, our data show that although isoflurane-dependent neural inertia is collapsed in *sss* mutants, neural inertia resulting from halothane-induced anesthesia is not. Taken alongside our rescue of anesthesia induction and neural inertia in *sss* mutants using different promoters, these data strongly suggest that different anesthetics utilize different arousal pathways to render animals unresponsive. That is, whereas anesthesia has often been treated as a whole-brain phenomenon, our data support actions for different anesthetics in specific circuits that govern arousal. Interestingly, of the mutations that been shown to affect general anesthesia, those with the biggest impact in flies (our data) and mammals [42] cause impairment of ion channel function. Whether these effects are due to loss of drug binding sites in the proteins affected by these mutations, or whether the resulting changes in membrane potential alter anesthetic efficacy [43] remains to be determined. Pharmacokinetics do not appear to be a factor, however, since at the EC_50_ for emergence in both flies and mammals, isoflurane concentrations are similar in controls and mutants that have altered neural inertia [7]. In any case, specific molecular and neuroanatomical changes clearly alter the state of anesthesia, thus supporting the idea that general anesthetics act on selective targets [11].

In summary, we have provided further evidence that neural inertia represents a barrier to changes in arousal state. We have also shown that this barrier can be genetically and anatomically dissected, and that it is distinguishable from the processes that control induction of anesthesia, at least when this state is studied with isoflurane. While these conclusions are based on studies of *Drosophila*, it is worth noting that we previously demonstrated genetic control over neural inertia in mammals as well, including mice deficient in noradrenaline production [7]. The commonality of neural inertia in such disparate organisms argues for conserved basic circuit design underlying control of arousal throughout evolution. It should be noted that although we have emphasized the possibility that circuit-based feedback mechanisms underlie bistability in our system, it is also possible that post-translational modifications contribute to this property.

In either case, the clinical importance of our findings is particularly notable for two reasons. First, our results confirm that the sensitivity to induction of anesthesia cannot be used to reliably predict how easily a patient will exit from the anesthesia state. Second, feedback and bistability may be impaired in coma or persistent vegetative states such that the neural inertial barrier separating waking from unconscious states is widened beyond the range of reversibility by normal physiological processes. The conservation of mechanisms underlying waking and anesthesia among distantly related phyla suggest that extension of our current work in *Drosophila* will continue to shed light on the genetic and anatomical processes underlying behavioral state stability, an issue of fundamental importance to both neuroscience and clinical medicine.

## Materials and Methods

### Fly stocks

All mutant and transgenic flies were outcrossed 4–7 times into an isogenic w^1118^ (iso31) background. Unless otherwise stated, controls for mutant animals were outcrossed siblings. GAL4 lines were generated or obtained as previously described [24], except for *Gr21a* and *nos*, which were obtained from the Bloomington Stock Center (Bloomington, IN). The *Sh^mns^* and *Sh^Df^* lines were obtained from D. Bushey, C. Cirelli and B. Ganetzky (University of Wisconsin), and *DAT^fmn^* flies were obtained from K. Kume (Kumamoto University). *na^e04385^* and *unc79^f03453^* were obtained from Bloomington, and *unc79^c04794^* was obtained from Exelixis (Harvard). *sss^P1^*, *sss^P2^*, and UAS-*sss* were described previously [24], [25].

### Behavioral assays

3–4 male and 5–8 female flies were combined on standard molasses-yeast-cornmeal food and allowed to mate at 21–23°C for 7–10 days. Adults were then discarded, and newly eclosing flies were collected over a 4 day period. 1–5 day-old females were loaded into 65×5 mm cylindrical tubes containing 5% sucrose and 2% agarose and entrained to a 12-hr∶12-hr light∶dark cycle for at least 2 d before being assayed for anesthetic sensitivity or sleep at 25°C using the *Drosophila* Activity Monitoring System (Trikinetics, Waltham, MA).

Anesthetics dissolved in air were delivered to flies in parallel, and final concentrations and flow rates were measured as previously described [7]. With flow rates set at 15 ml/min/tube, we calculate that gas concentrations inside our .75 ml tubes will reach equilibrium within 18 seconds. For anesthesia measurements, individual flies were exposed to increasing and then decreasing dosages of isoflurane using a previously described protocol [7]. The anesthetic endpoint that was used was immobility, with induction being defined as the lowest concentration at which movement ceased for five or more minutes, whereas emergence was defined as the highest concentration at which movement resumed.

Locomotor counts over 5 min periods for each individual fly were converted to a value of 1, signifying activity, or 0, indicating no movement. Flies that did not move for 15 minutes prior to the start of anesthesia or during the first 5 minutes at the lowest anesthetic dose were excluded from subsequent analysis. Flies that did not recover activity during the 24 hours following anesthesia were also excluded from analysis (<2% for the genetic background for all our experiments, w^1118^ iso31). Behavior was analyzed using custom software written in MATLAB (MathWorks, Natick, MA) where sleep was identified as periods of inactivity lasting at least 5 min [44]. Concentration-response curves were fit to the Hill equation using Prism 4 (GraphPad, La Jolla, CA), in which the top constant, degree of cooperativity (Hill coefficient) and EC_50_ were allowed to vary and only the bottom constant was constrained to zero.

Anesthetic experiments were conducted during the evening locomotor activity peak (ZT10:20 to ZT12:40). During this period, flies show consolidated activity and wakefulness. Responses to anesthetics are thus unlikely to be confounded by inactivity due to normal sleep. To calculate neural inertia, the area between the induction and emergence concentration-response curves was integrated over the range of the induction curve's EC_1_ to the emergence curve's EC_99_, as previously described [7]. Neural inertia for each set of induction and emergence curves is expressed as the mean ^±^ standard error.

To elicit sleep homeostasis, mechanical stimulation was applied to iso31 animals for 1 second every min for 24 hrs, ending at the last dose of applied isoflurane, using DAMS monitors mounted to a platform vortexer. Control iso31 animals received identical mechanical stimulation throughout dosing of anesthetic, but were not sleep-deprived prior to this time. Specifically, controls were placed on a vortexer with experimental animals beginning 15 minutes before the first dose of isoflurane and mechanically perturbed for 1 second every minute until the final dose of isoflurane at ZT12:40. Pilot studies were used to find the appropriate strength of mechanical stimulation to awaken sleeping but not anesthetized flies.

### Statistical analyses

Differences in neural inertia and sleep, as well as log(EC_50_)s for induction and emergence, were analyzed with one-way ANOVAs followed by Bonferroni correction for multiple comparisons or Student's t-tests (unpaired, two-tailed) where applicable.

## Supporting Information

Figure S1 Dose-response curves and neural inertia for isoflurane-dependent anesthesia in various *Sh* and *sss* mutants. (a) The hypomorphic *sss^P2^* mutation has no effect on induction (solid red) and only a mild but statistically insignificant effect on emergence (dashed red) compared to controls (solid and dashed black). *sss^P2^* does not significantly reduce neural inertia. (b) A deletion of part of the *Sh* locus (*Sh^Df^*) results in nearly coincident induction and emergence curves, leading to collapsed neural inertia (red). In contrast, the corresponding curves are well separated in sibling controls (black), resulting in significant neural inertia. (c,d) Induction is affected additively by *sss^P1^*and *Sh^mns^* (c) but not by *sss^P1^* and *Sh^Df^* (d). (e) The collapsed neural inertia phenotype is recessive for *sss^P1^* but dominant for *Sh^mns^* and *Sh^Df^* mutants. * p<.01 and ** p<.001 by one-way ANOVA with post-test Bonferroni correction.
(EPS)

Figure S2 (a) Sleep and activity bouts are fragmented in *na^e04385^* mutants. (b) Sleep in *unc79^c04794^* and *na^e04385^* mutants exceeds that of controls. ** p<.001 by two-tailed Mann-Whitney test. * p<.01 by one-way ANOVA with post-test Bonferroni correction.
(EPS)

Figure S3 Restoration of *sss* expression in different brain regions selectively rescues induction, emergence, and neural inertia. (a) Log EC_50_ values for anesthesia induction using control GAL4 driver/+ (white) and experimental GAL4 driver/+;UAS-*sss*/+ (black) animals with 22 different promoters, all in a *sss^P1^* mutant background. (b) Log EC_50_ values for anesthesia emergence using the same genotypes and labeling as in a. (c) Neural inertia for the same animals as in a and b. * p<.01 by one-way ANOVA with post-test Bonferroni correction.
(EPS)

Figure S4 (a) Dose-response curves for induction of and emergence from isoflurane-dependent anesthesia in *Clk^jrk^* (red) and sibling controls (black). (b) *DAT^fmn^* mutants have reduced daily sleep compared to sibling controls. ** p<.001 by unpaired t-test.
(EPS)

Table S1 All genotypes are listed in the left-hand column. Green labeling denotes progeny of Gal4 drivers crossed to wild-type (>+) or to UAS-*sss* (>UAS-sss), all within a *sss^P1^* mutant background. Corresponding values for log(EC_50_) and top constants of Hill fits to data points are shown for induction (red) and emergence (blue). Values for neural inertia and number of animals used (N) are shown in black.
(XLSX)
